# Supplementary material for: Gut Microbiota and Metabolites May Play a Crucial Role in Sea Cucumber Apostichopus japonicus Aestivation
Source: Microorganisms. 2023 Feb 7;11(2):416. doi: 10.3390/microorganisms11020416 (PMC9961660; doi:10.3390/microorganisms11020416)
Supplement: Supplementary file 1 [file microorganisms-11-00416-s001.zip › Figure S1.pdf]

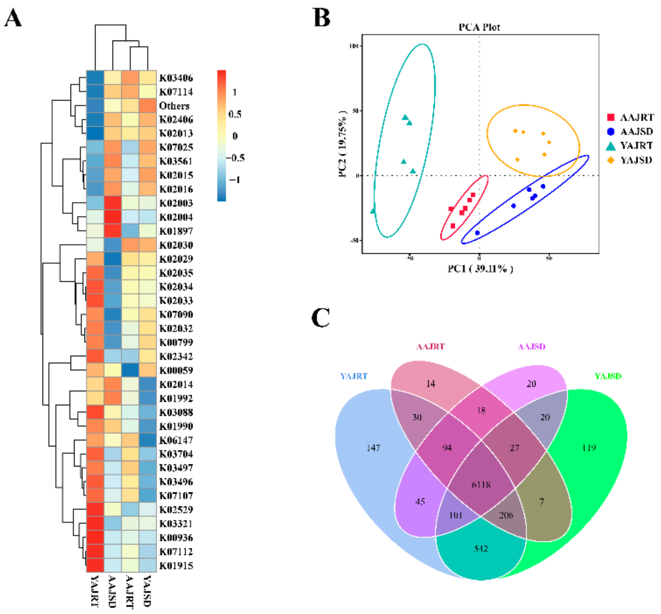

Figure S1. Functional prediction and association analysis of differential species among different groups

A. The results of functional prediction analysis of the KO database. The vertical direction is the sample information, the horizontal direction is the KO function annotation information, and the clustering tree on the left side of the figure is the function clustering tree. The value corresponding to the heatmap is the Z value obtained by normalizing the relative abundance of each row of functions. **B.** Principal component analysis (PCA) for dimensionality reduction (based on the KO database). The horizontal coordinate indicates the first principal component, and the percentage indicates the contribution of the first principal component to the sample variance. The vertical coordinate indicates the second principal component, and the percentage indicates the contribution of the second principal component to the sample variance. Each point in the graph indicates one sample, and the samples of the same group are represented using the same color. **C.** Quantitative analysis of shared and specific KO entries among different groups of gut microbiota. Each circle in the figure represents a group, and the number of overlapping circles represents the number of KO functions common to the group. The number without overlapping circles represents the number of KO functions specific to the group.
