# Supplementary material for: Gut Microbiota and Metabolites May Play a Crucial Role in Sea Cucumber Apostichopus japonicus Aestivation
Source: Microorganisms. 2023 Feb 7;11(2):416. doi: 10.3390/microorganisms11020416 (PMC9961660; doi:10.3390/microorganisms11020416)
Supplement: Supplementary file 1 [file Figure S2.pdf › Figure S2.pdf]

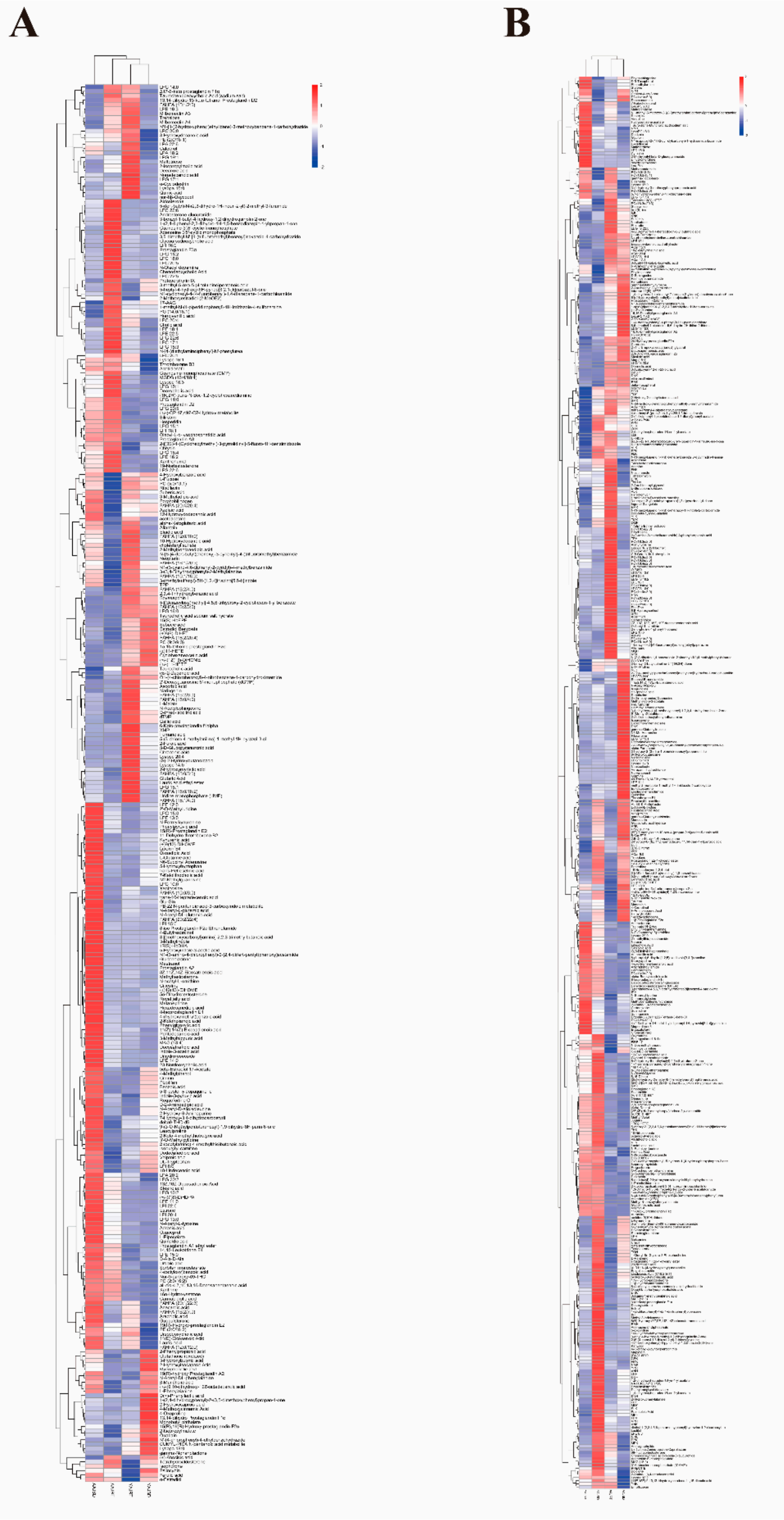

Figure S2. The results marked with the names of the differential metabolites among different groups
